# SARS-CoV-2 Pandemic: Not the First, Not the Last

**DOI:** 10.3390/microorganisms9020433

**Published:** 2021-02-19

**Authors:** Paolo Calistri, Nicola Decaro, Alessio Lorusso

**Affiliations:** 1 Istituto Zooprofilattico Sperimentale dell’Abruzzo e del Molise “G. Caporale”, 64100 Teramo, Italy; a.lorusso@izs.it; 2 Department of Veterinary Medicine, University of Bari, Valenzano, 70129 Bari, Italy; nicola.decaro@uniba.it

**Keywords:** SARS-CoV-2, coronavirus, bats, emerging infectious diseases

## Abstract

The common trait among the betacoronaviruses that emerged during the past two decades (the severe acute respiratory syndrome coronavirus—SARS-CoV, the Middle East respiratory syndrome coronavirus—MERS-CoV, and the recent SARS coronavirus 2—SARS-CoV-2) is their probable animal origin, all deriving from viruses present in bat species. Bats have arisen the attention of the scientific community as reservoir of emerging viruses, given their wide geographical distribution, their biological diversity (around 1400 species, 21 different families and over 200 genera), and their peculiar ecological and physiological characteristics which seem to facilitate them in harbouring a high viral diversity. Several human activities may enable the viral spill-over from bats to humans, such as deforestation, land-use changes, increased livestock grazing or intensive production of vegetal cultures. In addition, the globalization of trade and high global human mobility allow these viruses to be disseminated in few hours in many parts of the World. In order to avoid the emergence of new pandemic threats in the future we need to substantially change our global models of social and economic development, posing the conservation of biodiversity and the preservation of natural ecosystems as a pillar for the protection of global human health.

## 1. Introduction

In mid-December 2019 a novel coronavirus (CoV) of probable animal origin was identified in Wuhan, Hubei province of China, where pneumonia cases of unknown origin were observed [1]. This novel human CoV named lately SARS coronavirus 2 (SARS-CoV-2) [2,3] is the responsible for a severe, sometimes fatal disease referred to as COronaVIrus Disease 2019 (COVID-19) [4]. SARS-CoV-2 was able to spread to all continents in a few months, causing more than 85 million confirmed cases and 2 million deaths as of mid-January 2021. (https://www.worldometers.info/coronavirus/ (accessed on 19 February 2021)).

CoVs (family *Coronaviridae*, order *Nidovirales*) are enveloped, single-stranded RNA viruses, with the largest genome among RNA viruses (26–32 kb) [5]. Based on their genetic and antigenic relationships, the coronavirus study group of the International Committee for Taxonomy of Viruses (ICTV) classified CoVs into 4 genera, *Alpha-*, *Beta-*, *Gamma-* and *Deltacoronavirus* [6]. CoVs are known in several animal species, including horses, camels, cattle, swine, dogs, cats, rodents, birds, ferrets, minks, and various other wild animals [7]. Similar to other RNA viruses, coronaviruses can mutate at high rate and be able to adapt themselves and survive in various hosts and different ecosystems [8].

Over the past two decades, two additional highly pathogenic human betacoronaviruses have posed a threat of international concern for their capability of rapid spreading and human-to-human transmission: the severe acute respiratory syndrome coronavirus (SARS-CoV) and the Middle East respiratory syndrome coronavirus (MERS-CoV). SARS-CoV emerged at the end of 2002 in China, Guangdong province, and in 2003 it was able to spread to all continents through travellers with 8,096 confirmed cases and 774 deaths [9]. MERS-CoV was identified in September 2012 in Saudi Arabia in a patient with fever, cough, and dyspnoea [10]. Since the first case, MERS-CoV spread globally to 27 countries, causing 2519 laboratory-confirmed human cases and 866 deaths as of January 2020 [11]. The great majority of cases were reported in the Kingdom of Saudi Arabia (*n* = 2121) and in the other countries of the Arabian Peninsula. SARS-CoV-2 and SARS-CoV are 79% genetically identical, thus being included in the same viral species, namely *Severe acute respiratory syndrome-related coronavirus* (*SARSr-CoV*). MERS-CoV is slightly divergent and is included in a separate species, *Middle East respiratory syndrome-related coronavirus* [12,13]. Different viruses belonging to the *SARSr-CoV* species including RmYN02, RaTG13, bat-SL-CoVZC45, SL-CoVZXC21 have been detected in bats with a certain degree of nucleotide identity (93.3, 96.1, 87.6 and 87.4%, respectively) to SARS-CoV-2, respectively (Table 1) [12,14].

This strict relationship with strains detected in bats is a common trait between SARS-CoV-2 and the two other highly pathogenic CoVs, SARS-CoV and MERS-CoV, which are considered all deriving from viruses commonly present in bat species. The epidemiological links between bats and humans are not always known. In the cases of SARS-CoV and MERS-CoV, palm civets (*Paguma larvata*) and dromedary camels (*Camelus dromedarius*) are considered, respectively, the intermediate hosts between bats and humans [7]. In contrast, the possible intermediate host for SARS-CoV-2 has been not identified yet, although CoVs with a high similarity in the receptor binding motif of the spike protein have been detected in Malayan pangolins (*Manis javanica*) [14,15].

Besides human beings, SARS-CoV-2 proved to be able to infect and sometimes cause various respiratory and enteric clinical signs in cats, dogs, minks and wild *Felidae* [16,17,18,19,20] (Table 2 and Table 3).

This review aims at describing the current knowledge about the characteristics making bats so efficient as reservoirs of coronaviruses of public health concern and the factors driving the emergence and transfer to humans of these pathogens.

## 2. Bats as Reservoir of Emerging Viruses

Around 1400 bat species, belonging to 21 different bat families and over 200 genera, are known, populating all continents with the exception of Antarctica. Bats are the second mammalian order on Earth, after rodents, for the number of species included, accounting for approximately 22% of all mammal species [21]. Bats are gregarious animals and they can live in colonies with millions of individuals, frequently in caves or in roost over large fruit trees. Several bat species are migratory, even at long distances. In an experiment performed in Africa, straw-coloured fruit bats (*Eidolon helvum*) were tracked through a satellite system. These bats foraged as far as 59 km from the roost in a single evening, with one migrating individual being able to fly for 370 km in one night. Individuals were observed to travel for 2,518 km in around five months [22].

Bats have been identified as natural reservoir hosts for several emerging viruses, such as Marburg virus, Hendra virus, Sosuga virus and Nipah virus. To date, thousands of new bat-associated viral species from 28 viral families have been discovered. The great majority of these viruses are specific for bats with limited zoonotic potential, but other bat-associated viruses, such as coronaviruses, henipaviruses, lyssaviruses, filoviruses and orthoreoviruses [23], are of public and veterinary concern due to their capacity to emerge and spill-over to other wild and domestic animal species as well as to humans [21]. A strong evidence indicates that bats were involved in the emergence of a variety of viruses of major public health impact, such as Ebola viruses, SARS-CoV, MERS-CoV, and more recently SARS-CoV-2 [3,24,25,26].

Bats harbour a high viral diversity in comparison to other mammalian orders. A comprehensive analysis of mammalian host–virus relationships done by Olival et al. [27] demonstrated that bats harbour a significantly higher proportion of zoonotic viruses than all other mammalian orders.

The reasons beyond this viral richness in bats are not well known. The great number of bat species has been taken into consideration to explain this viral diversity hosted by bats. Recent studies, however, suggest that bats seem to have a certain degree of tolerance to viral infection, allowing the survival of the viruses and even developing an immune response, but not showing any clinical sign of illness. Several factors have been speculated to play a role in this “resistance” of bats to viral diseases. The particular bat’s metabolism, swinging from very low body temperatures during the hibernation period in winter at temperate climates to elevated body temperatures during flight, as an effect of the high-energy metabolic demands during the flight, would represent a mechanism hampering the viral replication [21]. However, several other hypotheses have been suggested, including the peculiar bat’s immune system that seems to not respond to viral infection with a strong activation of the inflammatory mechanisms, which represents the main cause of the clinical signs that can be observed in humans and laboratory animals [28,29].

Living in large colonies was considered a possible risk factor for the emergence of new viruses, although Turmelle and Olival [30] were not able to correlate host colony size with viral richness in bats. It must be considered, however, that the size of bat colonies can be extremely variable across species and can follow seasonal dynamics. Seasonal variation in colony size, therefore, may be more important than colony size alone in relation to the establishment of an efficient contact networks among animals, facilitating the emergence and persistence of viruses in bat populations [30].

In addition, the great adaptability of these animals to the environmental changes is demonstrated by the presence of fruit bats also in the urban context of major cities in Africa and Asia, which can be one of the factors possibly explaining the spill-over of viral infections to humans due to the increased human-bat interaction [31].

## 3. Drivers for Human Spill-Over

Around 70% of emerging viral diseases, and almost all recent pandemics, originated from animals, frequently from wildlife [32]. The emergence of these pathogens in wildlife and their progressive adaptation to domestic animals and/or human populations can be considered as the product of the natural evolution of infectious diseases, which characterises the human history from the very beginning. Karesh et al. [33] very well explained this principle: “Transmission of pathogens into human populations from other species is a natural product of our relation with animals and the environment. The emergence of zoonoses, both recent and historical, can be considered as a logical consequence of pathogen ecology and evolution, as microbes exploit new niches and adapt to new hosts”.

The emergence of new pathogens in wildlife is strictly related to species diversity and richness. However, all recent studies agree that, although natural areas characterised by rich biodiversity may represent a source for new pathogens, biodiversity loss frequently increases disease transmission. In fact, when the abundance and variety of hosts is reduced, pathogen may be more induced to find new niches. Hence, despite many unsolved questions, current evidence suggests that preserving intact ecosystems and their endemic biodiversity should reduce the emergence of infectious diseases in humans [34].

Disease spill-over to humans, however, is certainly influenced by anthropogenic changes such as deforestation, habitat fragmentation, land-use changes for the extraction of natural resources, increased livestock grazing or intensive production of vegetal cultures [32].

More than half (54%) of the World’s forests is in only five countries: Russian Federation, Brazil, Canada, the United States of America and China. Since 1990 the world has lost 178 million hectares of forest (an area about the size of Libya), with Africa experiencing the largest annual rate of net forest loss in 2010–2020 (about 3.9 million hectares) followed by South America (with around 2.6 million hectares) [35]. In Southeast Asia, for example, it was estimated that 45% of oil palm plantations came from areas that were forests in 1989. For South America, the same percentage was 31% [36]. The deforestation of rainforests poses particular threats to the global health because it increases significantly the probability for humans to come into contact with pathogens commonly circulating in wild animals, such as bats. For example, the emergence of Nipah virus in Malaysia in 1998 was linked to the intensification of pig production at the border of tropical forests where the fruit bat reservoirs live. In Bangladesh the repeated outbreaks of Nipah virus infection observed since 2001 were also associated to the consumption of date palm sap contaminated by bat’s faeces. In this case, the intensification in the cultivation of fruit trees for human consumption was the driver factor facilitating the contact between bats and humans [37].

**Figure 1:**
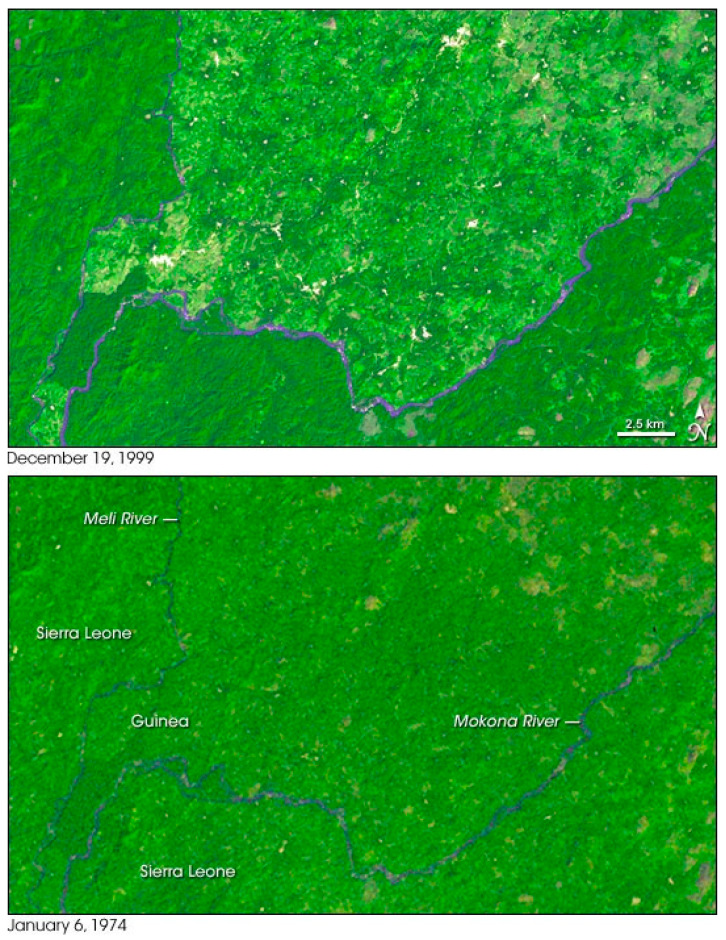
Deforestation in Guinea’s Parrot’s Beak Area. This pair of Landsat satellite images illustrates the deforestation that took place between 19 December 1999 (top), and 6 January 1974 (bottom). In 1974, deep green forests covered much more of the region than they did in 1999. Source: NASA’s Earth Observatory, https://earthobservatory.nasa.gov/images/6450/deforestation-in-guineas-parrots-beak-area (accessed on 19 February 2021).

The comparison between the satellite images of the jungles in Guinea bordering Liberia and Sierra Leone taken in 1974 with those of December 1999, showed a clear reduction of the green forest (Figure 1) [38]. At that time, Guinea, Liberia and Sierra Leone were affected by one of longest and cruel civil war ever observed in Africa, which eased in 2003. During the war a lot of people found refuge in the forest to escape from the violence of the war. After the end of the conflict, people hiding in the forest slowly returned to the cities, and all social and economic networks were progressively restored. Some authors consider the desegregation of the forest habitat followed by the return of the refugees to the cities as the main factors facilitating the explosion of the Ebola outbreak in 2013 in those countries [39]. As a partial confirmation of this hypothesis a serological study on people in eastern Sierra Leone, Liberia, and Guinea between 2006 and 2008 revealed that nearly 9% of tested persons had been exposed to Ebola [40].

Human contact with wildlife is increased also due to road building, establishment of new settlements, wildlife hunting and consumption of so called “bush-meat”. In developing countries all these phenomena are usually coupled with overcrowding, poor sanitary conditions, improper disposal of waste, and a lack of potable water, thus creating the best conditions for the explosion and transmissions of infectious agents accidentally introduced into the human population [32,33].

All these considerations are not new, but in the last decades we observed an exponential increase of threatening situations and, moreover, today the possibility for a new emerged pathogen to quickly spread all over the planet is incredibly greater than few years ago, due to globalization of commercial trade and increased global human mobility.

Human immunodeficiency virus (HIV) is believed to have emerged from non-human primates in the early 20th century, further spreading in the human population across Africa by truck routes and sexual practices [8]. Genetic studies of the virus suggest that the most recent common ancestor of the HIV dates back to around 1910 [41], and it took about 7 decades before to appear in United States.

In contrast, in less than two months from its recognition in China SARS-CoV-2 was able to reach several states of the European and American continents, thanks to the movements of asymptomatic or paucisymptomatic infected travellers.

## 4. Discussion

In past centuries, people were used to live under constant threats deriving by the regular occurrence of various infectious diseases. Epidemics of measles, cholera and other infectious diseases regularly occurred in several parts of the world. Various pandemic waves of plague hit the Old World: from 541 to around 750, spreading from Egypt to the Mediterranean (known as the Plague of Justinian), from around 1330 to about 1850, spreading from Central Asia to the Mediterranean and Europe, where it caused repeated outbreaks and the loss of around one third of the human population (so called Black Death). In 1918 the H1N1 pandemic flu (so called Spanish flu) spread from United States to Europe, causing 500 million of infections and at least 50 million deaths worldwide, being probably one of the factors that contributed to the end of the First World War, due to the difficulties for the countries to find healthy persons for their troops.

After the discovery of antibiotics, the progresses in the application of hygienic measures and the successes in vaccine preparation and production, humans were mostly freed from these nightmares, at least in the developed countries. The eradications of smallpox, reached in the eighties of the last century, and more recently (October 2019) of poliomyelitis due to wild poliovirus, were the most tangible results of the global efforts started several years ago with the “Health for all by the Year 2000” strategy, launched by the WHO with the Declaration of Alma-Ata in 1978 [42]. Despite these successes, diseases like cholera, rabies, tuberculosis and malaria are still present in many parts of the world, causing millions of deaths every year.

Two major emerging health issues, both driven by human activities, came under the spotlight in the last years: the increase of frequency and severity of antimicrobial resistance (AMR) phenomena in bacteria and the emergence of new pathogens with pandemic potential.

AMR is in the agenda of many governments, and several initiatives have been implemented to set global strategies for reducing the emergence of multi-resistant bacteria and containing the effects on the human health [43].

On the contrary, although the risks deriving from the emergence of new pathogens has been highlighted by the scientific community since at least two decades, only after the pandemic caused by the SARS-CoV-2 and its overwhelming consequences on the economics and health systems of hundreds of countries, the debate on the emerging diseases has left the scientific community to be a subject of political discussions.

In our overcrowded world of 7.8 billion people, human-driven environmental changes, coupled with inadequate public health mechanisms and socio-economic factors is heavily influencing our relationship with the wildlife, every days eroding new spaces to natural ecosystems and reducing biodiversity to a larger extent. In this context, the probability for human beings to come into contact with wild animal species, such as bats, is highly increased. These animals are known to be reservoirs of various viruses, especially RNA viruses, characterised by high mutation rates, which provide them with an extreme adaptability to new hosts and ecological niches. The frequency of spill-over from wild animals to humans, therefore, is more frequent than in the past and the recent emergence and spread of three novel coronaviruses (SARS-CoV, MERS-CoV and SARS-CoV-2 in 2002, 2012 and 2019 respectively), all originated from bats, clearly supports this scenario.

In the case of SARS-CoV-2, which is able to cause a wide range of clinical pictures in humans [44], it showed a high adaptability to new hosts has been clearly demonstrated by the outbreaks in mink farms, where human-to-mink viral transmissions were followed by the reverse mink-to-human infection [45], in an attempt of constituting new niches for the evolution and persistence of the virus. In such circumstances, the “classical” definition of zoonosis as “any disease or infection that is naturally transmissible from vertebrate animals to humans” (https://www.who.int/news-room/fact-sheets/detail/zoonoses (accessed on 19 February 2021)) shows its limits. A more holistic and ecological driven definition of zoonosis, such as “any infection shared between humans and other animals”, would better capture the complex interactions among hosts, parasites and ecological conditions.

The international organizations, well conscious of the global threats deriving from the emergence and spread of new pathogens, included these aspects in the so called Global Health Security Agenda and Sustainable Development Goals (SDGs) developed by the United Nations (UN). The Global Health Security Agenda (GHSA) is a group of 67 countries, international organizations, non-government organizations, and private sector companies that have come together to achieve the vision of a world safe and secure from global health threats posed by infectious diseases (https://ghsagenda.org/ (accessed on 19 February 2021)). The 2030 Agenda for Sustainable Development, adopted by all United Nations Member States in 2015, considers 17 Sustainable Development Goals (SDGs), as the pillar for action by all countries—developed and developing—in a global partnership (https://sustainabledevelopment.un.org/ (accessed on 19 February 2021)).

Mitigating drivers of disease emergence, which requires considerations of the multiple dimensions of socioeconomic development, is clearly part of the SDG 3 of the UN 2030 Agenda for Sustainable Development, which aims to “ensure healthy lives and promote wellbeing for all at all ages” [32].

It implies also a multidisciplinary approach to global health problems, through the application of the One Health concept, which should recognise the interaction between living beings, including men, animals and pathogens, sharing the same environment, as a unique dynamic system, in which the health of each component is inextricably interconnected and dependent with the others.

In the resolution of the third World Health Assembly on 19 May 2020 about COVID-19 response [46], World Health Organisation (WHO) was asked “to continue to work closely with the World Organisation for Animal Health (OIE), the Food and Agriculture Organization of the United Nations (FAO) and countries, as part of the One-Health Approach to identify the zoonotic source of the virus and the route of introduction to the human population, including the possible role of intermediate hosts, including through efforts such as scientific and collaborative field missions, which will enable targeted interventions and a research agenda to reduce the risk of similar events occurring, as well as to provide guidance on how to prevent infection with severe acute respiratory syndrome coronavirus 2 (SARS-COV2) in animals and humans and prevent the establishment of new zoonotic reservoirs, as well as to reduce further risks of emergence and transmission of zoonotic diseases.”

## 5. Conclusions

Nowadays, a new integrated One Health approach is reflecting this interdependence with a holistic view of the ecological system. The One Health approach can be defined as a collaborative and a multidisciplinary effort at local, national and global level to guarantee an optimal healthy status for humans, animals and environment [47].

If we do not change the global models of social and economic development into sustainable approaches from the ecological point of view, and if we do not reverse our paradigms, posing the conservation of biodiversity and the preservation of natural ecosystems as a pillar for the protection of global human health, we will continue to drain resources from the Earth, disrupting the integrity and the contiguity of natural habitats, thus continuously recreating all those circumstances that are the major drivers for the emergence and spread of pandemic infectious diseases. This is particularly important right now, when those countries that are coming out from strict lockdown periods due to SARS-CoV-2 pandemic are making great efforts in re-launching their economies, with the consequence of significantly increasing the demand of additional natural resources to be pumped in their economy. If we do not understand the lesson from SARS-CoV-2, if we are not conscious that a new approach to global of social and economic development is needed, SARS-CoV-2, which was not the first, will be not the last pandemic of this century.

## Figures and Tables

**Table 1:** Nucleotide sequence identity across the whole genome, individual ORFs coding for structural and accessory proteins, and receptor binding domain (RBD) (%) for SARS-CoV-2 compared with representative related beta-CoVs genomes. Adapted from [14].

Strain	Complete Genome	1ab	S	RBD	3a	E	M	6	7a	7b	8	N	10
**RmYN02**	93.3%	97.2%	71.9%	61.3%	96.4%	98.7%	94.8%	96.8%	96.2%	92.4%	45.8%	97.3%	99.1%
**RaTG13**	96.1%	96.5%	92.9%	85.3%	96.3%	99.6%	95.4%	98.4%	95.6%	99.2%	97.0%	96.9%	99.1%
**SL-CoVZC45**	87.6%	89.0%	75.1%	62.1%	87.8%	98.7%	93.4%	95.2%	88.8%	94.7%	88.5%	91.1%	99.1%
**SL-CoVZXC21**	87.4%	88.7%	74.6%	60.6%	88.9%	98.7%	93.4%	95.2%	89.1%	95.5%	88.5%	91.2%	/

**Table 2:** Pets and wild *Felidae* found infected by SARS-CoV-2 (source ProMed mail messages: https://promedmail.org/ (accessed on 19 February 2021)).

Month of Detection	Country	Type of Location	Animal Species	Observed Clinical Signs
Mar 2020	China (Hong Kong)	residential household	Cat (*Felis catus*)	No clinical signs
Mar 2020	China (Hong Kong)	residential household	Dog (*Canis lupus familiaris*)	No clinical signs
Mar 2020	Belgium	residential household	Cat (*Felis catus*)	Various clinical signs
Apr 2020	New York (US)	zoo	3 lions (*Panthera leo*), 2 Malayan tigers (*Panthera tigris jacksoni*), 2 Amur tigers (*Panthera tigris altaica*)	Coughing
Jul 2020	South Africa	zoo	Puma (*Puma concolor*)	No clinical signs
Oct 2020	Texas (US)	residential household	Cat (*Felis catus*)	No clinical signs
Oct 2020	Utah (US)	wild	American mink (*Neovison vison*)	
Oct 2020	Tennessee (US)	zoo	Malayan tiger (*Panthera tigris jacksoni*)	3 tigers showing mild coughing, lethargy, and decrease in appetite.
Nov 2020	Slovenia	residential household	Ferret (*Mustela putorius furo*)	Gastrointestinal clinical signs
Nov 2020	Florida (US)	residential household	Dog (*Canis lupus familiaris*)	History of respiratory issues
Nov 2020	Pennsylvania (US)	residential household	Dog (*Canis lupus familiaris*)	History of respiratory issues
Nov 2020	Texas (US)	residential household	Cat (*Felis catus*)	3 cats: 2 with no clinical signs and 1 showing tremors, vomiting, and sneezing
Nov 2020	Kentucky (US)	zoo	Snow leopards (*Panthera uncia*)	mild respiratory clinical signs, including dry cough or wheeze.
Nov 2020	Wisconsin (US)	residential household	Cat (*Felis catus*)	clinical signs of lethargy, sinus congestion, wheezing, sneezing, and nasal discharge
Dec 2020	Kansas (US)	residential household	Dog (*Canis lupus familiaris*)	nasal discharge
Dec 2020	Spain	zoo	Lion (*Panthera leo*)	4 lions with mild clinical signs
Jan 2021	San Diego (US)	Zoo	Gorillas (*Gorilla gorilla*)	2 gorillas with coughing

**Table 3:** Mink (*Neovison vison*) farms found infected by SARS-CoV-2 (source ProMed mail messages: https://promedmail.org/ (accessed on 19 February 2021)).

Month of First Detection	Country
Apr 2020	the Netherlands
Jun 2020	Denmark
Jun 2020	Spain
Aug 2020	Utah (US)
Oct 2020	Italy
Oct 2020	Sweden
Nov 2020	France
Nov 2020	Greece
Nov 2020	Lithuania
Nov 2020	Poland
Nov 2020	Wisconsin (US)
Nov 2020	Canada

## Data Availability

No new data were created or analyzed in this study. Data sharing is not applicable to this article.
